# Biographical Sketch of the Late Mr. Pearson

**Published:** 1826-07

**Authors:** 


### *Biographical Sketch of the late* Mr. Pearson. By a Correspondent.

Mr. John Pearson was born at York, on the 3d January, 1758. At the age of sixteen, he began his professional education at Morpeth in Northumberland, where he resided three years. In the month of June, 1777, he removed to Leeds, and placed himself under the tuition of Mr. Hey, who even at that time possessed considerable eminence in that part of the country. The opportunities of practice which Mr. Pearson enjoyed while in this situation were very great, especially in the surgery of accidents and operations; in which respect he always described the Leeds Infirmary as superior to the greater part of the London hospitals. There is also no doubt that the sound judgment and laborious perseverance which he witnessed in Mr. Hey, contributed much to strengthen his intellectual faculties, and confirm him in habits of industry. In the year 1780, he came to London, and continued his studies at St. George's Hospital, and in the anatomical school of Dr. Hunter. It was his original intention to have remained in town only for such a length of time as would have enabled him to become a member of the College, and then to have returned into the country, and established himself either at York or at Hull; but he was induced to form other plans, by events which could not have been anticipated.

In the year 1781, the Lock Hospital had fallen into serious difficulties, chiefly from the utter inefficiency of the medical establishment, one of the surgeons being constantly resident abroad, and the other incapable, from age and infirmities, of discharging his duties. At this juncture it so happened that Mr. Pearson became house-surgeon to the hospital, and the assiduity and talents he displayed in that situation so attracted the notice of the governors, that, in April 1782, they constituted him surgeon. Similar reasons induced the founders of the Public Dispensary in Carey-street to select him as surgeon to that institution, which was established about the same period. These two occurrences induced him to alter his plan, and to take up his residence in London; and it soon appeared that his professional prospects would not suffer by the change.

It was his lot, at an early period, to enjoy the society of some individuals of great talents and high scientific distinction, who have been long numbered with the dead. His mind was thus drawn to the higher paths of philosophy, especially to the works of Lord Bacon, on which he bestowed much labour, and acquired a most thorough and intimate acquaintance with them. These studies he always considered as mainly conducive to his subsequent progress in professional knowledge, and to the distinction which followed it: nor did he ever fail to recommend a similar course to those in whose success he was particularly interested. He was desirous of carrying the lessons he had thus learnt into his own medical pursuits, and of founding that science, if possible, on a more solid and philosophical basis. With this view he always prefaced his lectures on Surgery by lectures on Physiology, considering the latter as inseparable from the former, and esteeming it absolutely essential to the comprehension of the first principles of medical treatment. Such was the mode of study which was first introduced into this country by Mr. Hunter, but which his contemporaries, and even his successors, have been slow in imitating. In general, physiology is used rather to give clearness and interest to the study of anatomy, than in its higher and more useful application as an introduction to medicine and surgery. While this is the case, medicine can scarcely be called a science: it can be little more than a collection of facts, arranged with more or less attention to method.

The physiological lectures of Mr. Pearson were distinguished by the quantity of matter which they contained, and which often, it must be confessed, made them beyond the comprehension of the ordinary class of medical students. The conclusions were remarkably sound, and have, in almost all instances, been confirmed by subsequent discoveries. His lectures on surgery, in the same way, contained an immense mass of knowledge, the general result of all his reading and experience; which he augmented from year to year by the addition of every important remark that he met with, and every new conclusion that he arrived at in practice. The highest merit undoubtedly belongs to his lectures on Syphilis, which are less marked by erudition, but display, in an eminent degree, the power which he possessed of arranging his knowledge, and treating, without confusion or perplexity, a most extensive and complicated subject. They were written later than his other lectures, having been originally delivered in 1807 to military cadets, at the desire of the Army Medical Board, and subsequently repeated, with some additions, to a small class of pupils in the year 1811. They have little reference to the theory of the disease, which Mr. Pearson always felt to be extremely obscure and uncertain: their value is entirely practical, and, from the accuracy with which symptoms are described, and the excellence of the rules of treatment, they form the best practical treatise which exists on venereal disorders; and it may be added, that recent experiments have in no degree affected their utility or truth, which would be felt the more strongly by each individual, in proportion as his experience in such cases is more extensive.

The principles which are inculcated in these lectures were exemplified in the Lock Hospital, where the practice of Mr. Pearson was remarkably systematic. He held the office of surgeon to that institution upwards of thirty-six years, during the early part of which period he had the whole of the patients under his sole management. Other avocations, joined to ill health, latterly prevented him from bestowing that attention which he wished on its duties, and he finally resigned it in the year 1818. Similar considerations had induced him to relinquish the Public Dispensary in the year 1809.

It was the intention of Mr. Pearson at one period to have written much, and in detail, on the science of Surgery, reduced to the principles on which it is founded; and undoubtedly the philosophical character of his mind peculiarly qualified him for the execution of such a project. He had actually made some preparation for its accomplishment on an extensive scale, but he was compelled to abandon his intention by a long course of ill health, which rendered him incapable of continued application; while a certain fastidiousness of judgment, almost inseparable from extensive reading, gradually crept upon him, and rendered him less satisfied with his productions. Of the whole which he projected, we have nothing but the "Principles of Surgery," which are little more than a very short syllabus of a part of his lectures. He has left, besides, a small tract on Cancer, and also "Observations on the Effect of various Articles of the Materia Medica in the Cure of Lues Venerea," in which he has interspersed some valuable remarks on the different forms of the disease, and on the effects of mercurial remedies. These, with the Life of Mr. Hey, a single paper in the Philosophical Transactions on the *Ibis*, and a few communications to periodical publications, constitute the whole which he has given to the press, and afford very limited means of estimating either the extent of his knowledge, or the strength and compass of his intellectual powers. A just opinion of these can only be formed by those who were intimately acquainted with him.

On the 6th of May, he was seized with a sudden and violent attack of fever, under which he sunk rapidly, and expired at the end of six days, on the 12th of the same month.

